# Hypoxia exacerbates the malignant transformation of gastric epithelial cells induced by long-term *H. pylori* infection

**DOI:** 10.1128/spectrum.00311-24

**Published:** 2024-06-25

**Authors:** Yang He, Xiulin Zhang, Xiaolu Zhang, Bo Fu, Jin Xing, Rui Fu, Jianyi Lv, Meng Guo, Xueyun Huo, Xin Liu, Jing Lu, Lixue Cao, Xiaoyan Du, Zhongming Ge, Zhenwen Chen, Xuancheng Lu, Changlong Li

**Affiliations:** 1 Beijing Key Laboratory of Cancer Invasion & Metastasis Research, Department of Medical Genetics and Developmental Biology, School of Basic Medical Science, Laboratory for Clinical Medicine, Capital Medical University, Beijing, China; 2 School of Nursing, Dalian Medical University, Dalian, China; 3 Fuwai Hospital, Chinese Academy of Medical Sciences and Peking Union Medical College, Beijing, China; 4 Institute for Laboratory Animal Resources, National Institutes for Food and Drug Control, Beijing, China; 5 Division of Comparative Medicine, Massachusetts Institute of Technology, Cambridge, Massachusetts, USA; 6 National Key Laboratory of Intelligent Tracking and Forecasting for Infectious Diseases, Chinese Center for Disease Control and Prevention, Beijing, China; Beijing Institute of Genomics, Beijing, China

**Keywords:** *Helicobacter pylori*, hypoxia, gastric epithelial cell, carcinogenesis, gastric cancer

## Abstract

*Helicobacter pylori* is a microaerophilic Gram-negative bacterium that resides in the human stomach and is classified as a class I carcinogen for gastric cancer. Numerous studies have demonstrated that *H. pylori* infection plays a role in regulating the function of host cells, thereby contributing to the malignant transformation of these cells. However, *H. pylori* infection is a chronic process, and short-term cellular experiments may not provide a comprehensive understanding of the *in vivo* situation, especially when considering the lower oxygen levels in the human stomach. In this study, we aimed to investigate the mechanisms underlying gastric cell dysfunction after prolonged exposure to *H. pylori* under hypoxic conditions. We conducted a co-culture experiment using the gastric cell line GES-1 and *H. pylori* for 30 generations under intermittent hypoxic conditions. By closely monitoring cell proliferation, migration, invasion, autophagy, and apoptosis, we revealed that sustained *H. pylori* stimulation under hypoxic conditions significantly influences the function of GES-1 cells. This stimulation induces epithelial-mesenchymal transition and contributes to the propensity for malignant transformation of gastric cells. To confirm the *in vitro* results, we conducted an experiment involving Mongolian gerbils infected with *H. pylori* for 85 weeks. All the results strongly suggest that the Nod1 receptor signaling pathway plays a crucial role in *H. pylori*-related apoptosis and autophagy. In summary, continuous stimulation by *H. pylori* affects the functioning of gastric cells through the Nod1 receptor signaling pathway, increasing the likelihood of cell carcinogenesis. The presence of hypoxic conditions further exacerbates this process.

**IMPORTANCE:**

Deciphering the collaborative effects of *Helicobacter pylori* infection on gastric epithelial cell function is key to unraveling the development mechanisms of gastric cancer. Prior research has solely examined the outcomes of short-term *H. pylori* stimulation on gastric epithelial cells under aerobic conditions, neglecting the bacterium’s nature as a microaerophilic organism that leads to cancer following prolonged stomach colonization. This study mimics a more genuine *in vivo* infection scenario by repeatedly exposing gastric epithelial cells to *H. pylori* under hypoxic conditions for up to 30 generations. The results show that chronic exposure to *H. pylori* in hypoxia substantially increases cell migration, invasion, and epithelial-mesenchymal transition, while suppressing autophagy and apoptosis. This highlights the significance of hypoxic conditions in intensifying the carcinogenic impact of *H. pylori* infection. By accurately replicating the *in vivo* gastric environment, this study enhances our comprehension of *H. pylori*’s pathogenic mechanisms in gastric cancer.

## INTRODUCTION

Gastric cancer is a significant global health concern, ranking as the fifth most common cancer and the second leading cause of cancer-related deaths worldwide. In 2020, there were over 1 million new cases of gastric cancer and approximately 770,000 related deaths, posing a serious threat to the well-being of individuals across the globe (1). The development of gastric cancer is influenced by various factors, and one notable and preventable cause is the infection of *Helicobacter pylori*. *H. pylori* is a Gram-negative bacterium that thrives in the stomach’s microaerophilic environment. It is the most prevalent bacterial infection worldwide and is closely associated with gastritis, gastric ulcer, and even gastric cancer. In fact, *H. pylori* has been classified as a class I carcinogen for human gastric cancer (2–5).

During the colonization in the mucosa, *H. pylori* consistently releases a diverse range of bacterial toxins, including cytotoxin-associated gene product A (CagA), vacuolating cytotoxin A (VacA), lipopolysaccharide, and peptidoglycan (6–11). Additionally, *H. pylori* secretes outer membrane vesicles (OMV) containing various bacterial toxins and antigens (11). These components actively participate in modulating crucial functions of host cells, such as proliferation, autophagy, apoptosis, migration, and invasion. As a result, they promote cellular malignant transformation and contribute to gastric carcinogenesis (11–13). Our previous investigation unveiled the ability of *H. pylori* to deliver CagA toxin protein to gastric epithelial cells via OMV in the lysate, thereby inducing cellular transformation toward cancer through the Nod1 pathway. However, the precise underlying mechanism by which *H. pylori* triggers gastric cancer remains to be fully elucidated.

Despite numerous studies on *H. pylori’s* pathogenicity, previous research often used short-term stimulation to mimic *in vivo* infection. However, *H. pylori* infection is a chronic process, and short-term cellular experiments don’t replicate the true *in vivo* scenario. Additionally, the oxygenation characteristics of human gastrointestinal tract are highly distinctive (14). Oxygen levels vary, with oxygen partial pressure (pO_2_) ranging from 13.33 to 14.66 kPa in lung alveoli, 7.73 kPa in the stomach, and an anaerobic environment in the gastrointestinal lumen (14–17). Previous studies predominantly operated under normal oxygen partial pressure, which was higher than the stomach environment, thus failing to replicate the true impact of *H. pylori* infection on gastric epithelial cell function under realistic conditions.

In this study, we artificially created a hypoxic environment to simulate the authentic gastric conditions during continuous *H. pylori* infection. GES-1 cells were co-incubated with *H. pylori* under intermittent hypoxia conditions for 30 generations. Simultaneously, a control cell line was established through intermittent hypoxia culture of GES-1 cells for 30 generations. We further investigated the effects of long-term *H. pylori* stimulation under hypoxic conditions on cell functions and the Nod1 signaling pathway. Our findings demonstrate that the hypoxic gastric environment exacerbates the malignant transformation of gastric epithelial cells induced by sustained *H. pylori* infection.

## MATERIALS AND METHODS

### Bacterial strain and bacterial culture

The *H. pylori* strain ATCC 43504 (cagA^+^, vacA^+^) was obtained from the National Institutes for Food and Drug Control. *H. pylori* was cultivated on Campylobacter Agar Base (Karmali) plates (OXOID, UK, CM0935) supplemented with 5% sterile and defibrinated sheep blood (MRC, China, CCS30037.01) at 37°C under microaerophilic conditions (5% O_2_, 10% CO_2_, and 85% N_2_) for 48 hours.

### Cell lines and cell culture

The human normal gastric epithelial cell line GES-1 was procured from Ningbo Mingzhou Biotechnology Co. Ltd. (China, MZ-0779). Cells were cultured in Dulbecco's modified eagle medium (DMEM, Corning, USA, 10-013-CVR) supplemented with 10% fetal bovine serum (FBS) (PAN, Germany, P30-3302) and 1% penicillin/streptomycin binary antibody solution (KeyGen BioTECH, China, KGY0023) in a humidified environment with 20% O_2_, 5% CO_2_ at 37°C.

### Cell treatment

In the experimental group (Hp_30_-GES-1), GES-1 cells were co-cultured with *H. pylori* for 30 consecutive generations under microaerophilic conditions (5% O_2_, 10% CO_2_, and 85% N_2_). *H. pylori* strain ATCC43504 (6 × 10^8 CFU) was added to GES-1 cells at a bacteria-to-cell ratio of 1,000:1. After a 24-hour co-culture with *H. pylori* under hypoxic conditions, the cells underwent a PBS wash to remove the bacteria. Subsequently, the cells were cultured for another 24 hours under aerobic conditions (20% O_2_, 5% CO_2_, and 75% N_2_) in a sterile environment to restore their vitality, marking one generation in continuous culture. To assess the impact of continuous hypoxia culture on cells, a hypoxia control group (Hy_30_-GES-1) was established. The Hy_30_-GES-1 cells were cultured for 30 generations under microoxygen conditions of 5% O_2_, 10% CO_2_, and 85% N_2_. The culture method of the Hy_30_-GES-1 cell line mirrors that of Hp_30_-GES-1, except it does not involve the introduction of *H. pylori* throughout the entire process. To serve as a control, untreated wild-type cells (B-GES-1) were cultured for 30 consecutive generations, where each generation represented a 48hour culture period.

### Detection of *H. pylori* infection activity

GES-1 cells were co-cultured with *H. pylori* for 24 hours, and the cell supernatant was collected. After centrifugation, the supernatant was discarded, and the precipitated bacteria was resuspended in PBS. A 200 µL bacterial suspension was inoculated on Campylobacter Agar Base (Karmali) plates and incubated for 48–72 hours in a microaerobic environment at 37°C. Subsequently, bacterial identification was conducted through Gram staining microscopy, catalase testing, urease testing, and oxidase testing to confirm *H. pylori*.

### Colony formation assay

B-GES-1, Hy_30_-GES-1, and Hp_30_-GES-1 cells (500 cells/well) were plated in new 6-well plates and cultured in DMEM medium containing 10% FBS under 5% CO_2_ at 37°C. After 14 days, the cells were fixed and stained with 0.1% crystal violet. Visible colonies were counted using ImageJ software, with each well assessed in triplicate.

### CCK-8 assay

B-GES-1, Hy_30_-GES-1, and Hp_30_-GES-1 cells were counted and plated in 96-well plates at a density of 3,000 cells per well. After cell attachment, 10 µL of CCK-8 reagent (Vazyme, China, A311-01) was added to each well at 0 hours, 12 hours, 24 hours, and 48 hours and incubated at 37°C for 2 hours. The absorbance at a wavelength of 450 nm was measured using a microplate reader, and these values were used to generate cell proliferation curves. Each treatment was performed in quintuplicate

### Wound healing assay

C-GES-1, Hy_30_-GES-1, and Hp_30_-GES-1 cells were seeded in 6-well plates and cultured in DMEM medium containing 10% FBS. When the cells reached 90% confluence, a wound was created by discarding the medium, followed by washing the cells twice with PBS to remove any floating cells.

### Transwell migration and invasion assay

For the migration assay, B-GES-1, Hy_30_-GES-1, and Hp_30_-GES-1 cells (3 × 10^4^ cells in 200 µL) were placed in the upper chamber of a Transwell migration chamber with serum-free medium. Medium containing 10% FBS was added to the lower chamber. After 24 hours of incubation, the cells were fixed, stained with 0.1% crystal violet, and then counted. The assays were performed in triplicate.

For the invasion assay, 3 × 10^4^ cells were seeded into the upper chamber of a Transwell invasion chamber with serum-free media, while medium containing 10% FBS was added to the lower chamber. After a 24-hour incubation, the cells were fixed, stained with 0.1% crystal violet, and counted. The assays were performed in triplicate.

### mCherry-EGFP-LC3 transfection

B-GES-1, Hy30-GES-1, and Hp30-GES-1 cell suspensions with a density of 2.5 × 10^4^ cells/mL were prepared in complete medium and added to a six-well plate. After cell attachment, the old medium was discarded, and the cells were washed with PBS. Medium containing a 50-mL titer of 10^8^ TU/mL mCherry-EGFP-LC3 lentivirus (Hanbio Biotechnology Co. Ltd., Shanghai, China) and 8 mg/mL Polybrene was added to the culture. After 48 hours of infection, the fluorescence expression of cells was observed using fluorescence microscopy. When the cell infection efficiency reached approximately 80%, the cells were cultured in medium containing *H. pylori* for an additional 24 hours. The images of mCherry-EGFP-LC3-transfected cells were captured using laser scanning confocal microscopy.

### Apoptosis assay

Flow cytometry was employed to detect the effects of *H. pylori* on the apoptosis of B-GES-1, Hy_30_-GES-1, and Hp_30_-GES-1 cells using the Annexin V-PE/7-AAD Apoptosis Detection Kit (Vazyme, China, A213-01) according to the manufacturer’s instructions. The rate of apoptosis was analyzed using the LSR Fortessa Flow Cytometer at 488 nm.

### Reverse transcriptase polymerase chain reaction

Total RNA was isolated from B-GES-1, Hy_30_-GES-1, and Hp_30_-GES-1 cells using TRIzol Reagent (Vazyme, China, R401-01). Single-stranded DNA was synthesized from 1 mg total RNA using reverse transcriptase-bound oligonucleotide (DT) primers. Each cDNA sample (2 mL) was subjected to reverse transcriptase polymerase chain reaction amplification using specific primers as detailed in Table S1. The data were collected and analyzed. The values were compared with the experimental controls after normalization to those of GAPDH.

### Western blot

Following treatment with *H. pylori*, B-GES-1, Hy_3_0-GES-1, and Hp_30_-GES-1 cells were lysed using Radio Immunoprecipitation Assay (RIPA) Lysis Buffer (Solarbio, China, R0010) on ice for 30 minutes. The cell lysate was then centrifuged at 13,400 × *g* at 4°C for 15 minutes. The supernatants were collected, and the protein concentration was determined using a BCA protein kit (Thermo Scientific, USA, A53225). The lysate was mixed with PBS and 5× SDS loading buffer (ROBY, China, RBU114-2) and heated at 99°C for 10 minutes. Western blots were conducted using 8% or 10% SDS-polyacrylamide gel electrophoresis gel, and the protein samples were transferred onto polyvinylidene fluoride (PVDF) membranes (Merck Millipore, USA, ISEQ00010). PVDF membranes were blocked with 5% skim milk (BD, USA, 232100) and incubated overnight at 4°C with rabbit primary antibodies. This was followed by incubation with a secondary antibody (Solarbio, China, SE134, diluted 1:5,000 for western blot) for 1 hour. β-Actin (HuaBio, China, R1207-1, diluted 1:1,000 for western blot) was used as a loading control. The primary antibodies used in this study were as follows: LC3B-II (CST, USA 2775, diluted 1:1,000), p62 (Abcam, UK, ab56416, diluted 1:1,000), Nod1 (CST, USA, 3545S, diluted 1:1,000), RIP2 (Abcam, UK, ab8428, diluted 1:1,000), p-ERK1/2 (CST, USA, 4370S, diluted 1:1,000), ERK1/2 (CST, USA, 4695, diluted 1:1,000), FOXO4 (CST, USA, 9472, diluted 1:1,000), p-IKKA (Abcam, UK, ab38515, diluted 1:1,000), IKKA (Abcam, UK, ab32041, diluted 1:1,000), N-cadherin (proteintech, USA, 22018-1-AP, diluted 1:2,000), E-cadherin (proteintech, USA, 20874-1-AP, diluted 1:5,000), ZO-1 (Abcam, UK, ab216880, diluted 1:1,000), Snail (CST, USA, 3879T, diluted 1:1,000), and p-PI3K (CST, USA, 4228T, diluted 1:1,000).

### *H. pylori-*infected Mongolian gerbil model

Five Mongolian gerbils weighing 60–80 g were used to establish the *in vivo H. pylori* infection model, with five *H*. *pylori*-negative gerbils as a control group. Gerbils were obtained from the Capital Medical University and housed in secondary biosafety laboratories at the Chinese Center for Disease Control and Prevention. They were kept in standard plastic cages with a 12-hour light/dark cycle and provided with free access to food and water throughout the experiments. Gerbils aged 6–8 weeks were orally infected with *H. pylori* ATCC 43504 strain solution using a 0.5 mL dose of 2 × 10^9^ CFU/mL. Prior to challenge, the gerbils were fasted for 12 hours. Oral gavage was performed five times at 48-hour intervals. Before euthanasia, the colonization of *H. pylori* in gerbils was confirmed through ^13^C urea breath test and PCR detection. After 90 weeks of infection, the gerbils were euthanized, and gastric mucosa samples were collected for H&E staining and immunohistochemistry (IHC).

### Immunohistochemistry

Stomach sections were incubated overnight at 4°C with rabbit monoclonal Nod1 (CST, USA, 3545S, diluted 1:1,000 for IHC) and RIP2 (Abcam, UK, ab8428, diluted 1:1,000 for IHC). Subsequently, the sections were incubated with the corresponding biotinylated secondary antibody. Cell nuclei were counterstained with hematoxylin, and the samples were dehydrated in a gradient series, vitrified with dimethylbenzene, and finally mounted with neutral balsam.

### Statistical analysis

All statistical analyses were performed using SPSS v19.0 software. Data were presented as mean ± standard deviation. Independent sample *t*-test and one-way analysis of variance (ANOVA) were employed to determine the significance of differences between the results. A *P* value of less than 0.05 was considered statistically significant.

## RESULTS

### Establishment of the GES-1 cell line with *H. pylori* continuous infection under hypoxic conditions

**Fig 1:**
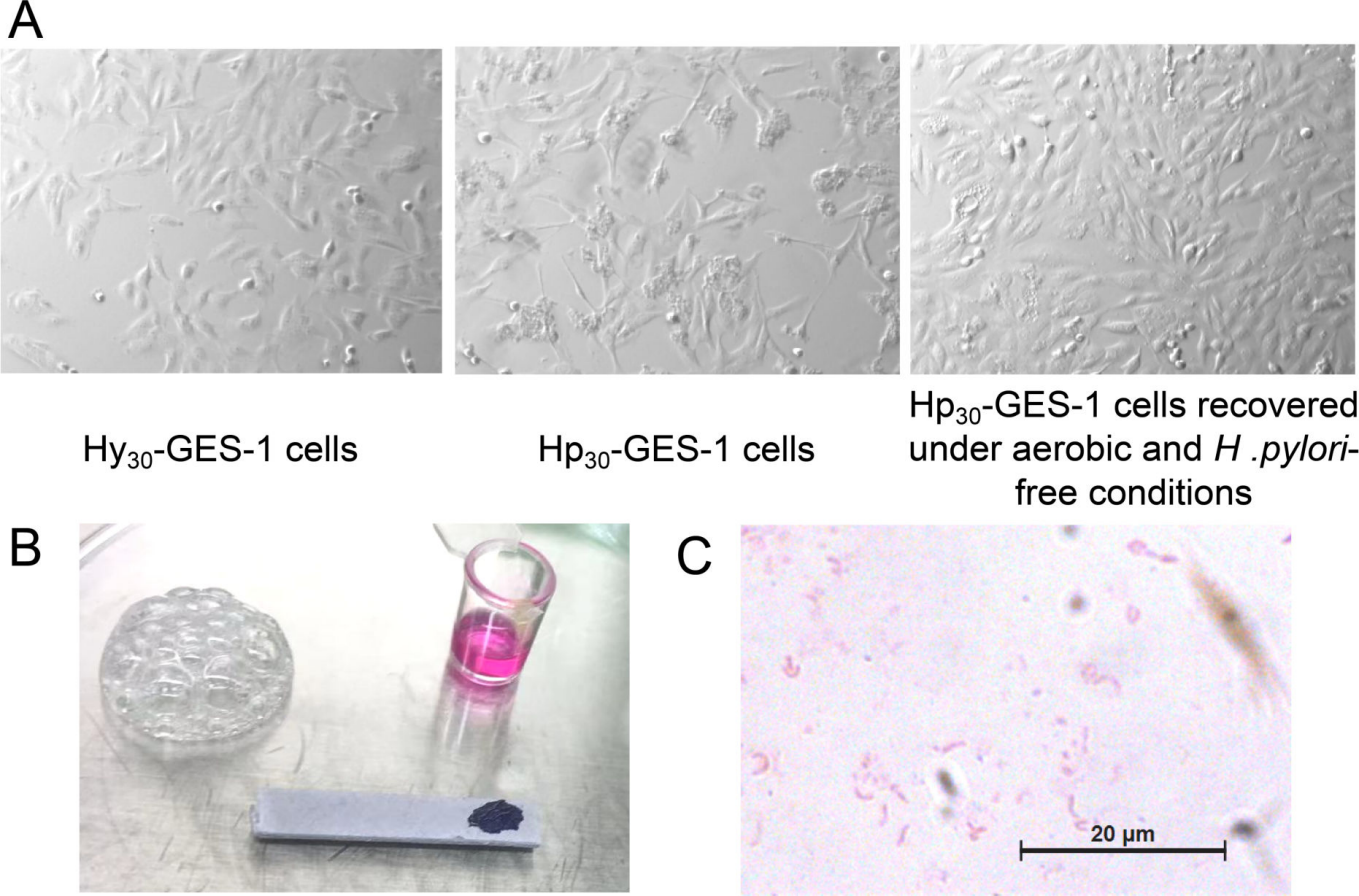
Establishment and detection of GES-1 cell line with sustained exposure to *H. pylori* under hypoxic condition. (**A**) Microscopic imaging systems were used to observe cell morphology of Hp_30_-GES-1 and Hy_30_-GES-1 cells. (**B and C**) The culture supernatant of GES-1 cells co-cultured with *H. pylori* under hypoxia for 24 hours was collected for bacterial culture, and *H. pylori* strains were successfully cultivated. Catalase test, urease test, and oxidase test (**B**) and Gram staining microscopy (**C**) were performed to confirm the activity of *H. pylori*. Hy30-GES-1 cells: GES-1 cells cultured for 30 generations under microoxygen conditions. Hp30-GES-1: GES-1 cells co-cultured with *H. pylori* for 30 consecutive generations under microaerophilic conditions.

During the establishment of gastric epithelial cell lines continuously exposed to *H. pylori* (Hp_30_-GES-1) or not (Hy_30_-GES-1) under hypoxic conditions, we employed two methods to verify *H. pylori* infection. First, microscopic imaging systems were used to observe cell morphology. GES-1 cells exhibited a typical hummingbird phenotype after co-culturing with *H. pylori* for 24 hours under hypoxic conditions, while cells cultured solely under hypoxia and the control group did not display this phenotype (Fig. 1A). Second, after co-culturing cells with *H. pylori* under hypoxia for 24 hours, the culture supernatant was collected for bacterial culture, and *H. pylori* strains were successfully cultivated. The existence of live bacteria was confirmed by morphology strain, Gram staining microscopy, catalase test, urease test, and oxidase test (Fig. 1B and C). All the results confirm that active *H. pylori* continuously attack GES-1 cells during the establishment of the cell lines.

### Sustained exposure to *H. pylori* under hypoxic conditions promotes cell proliferation

**Fig 2:**
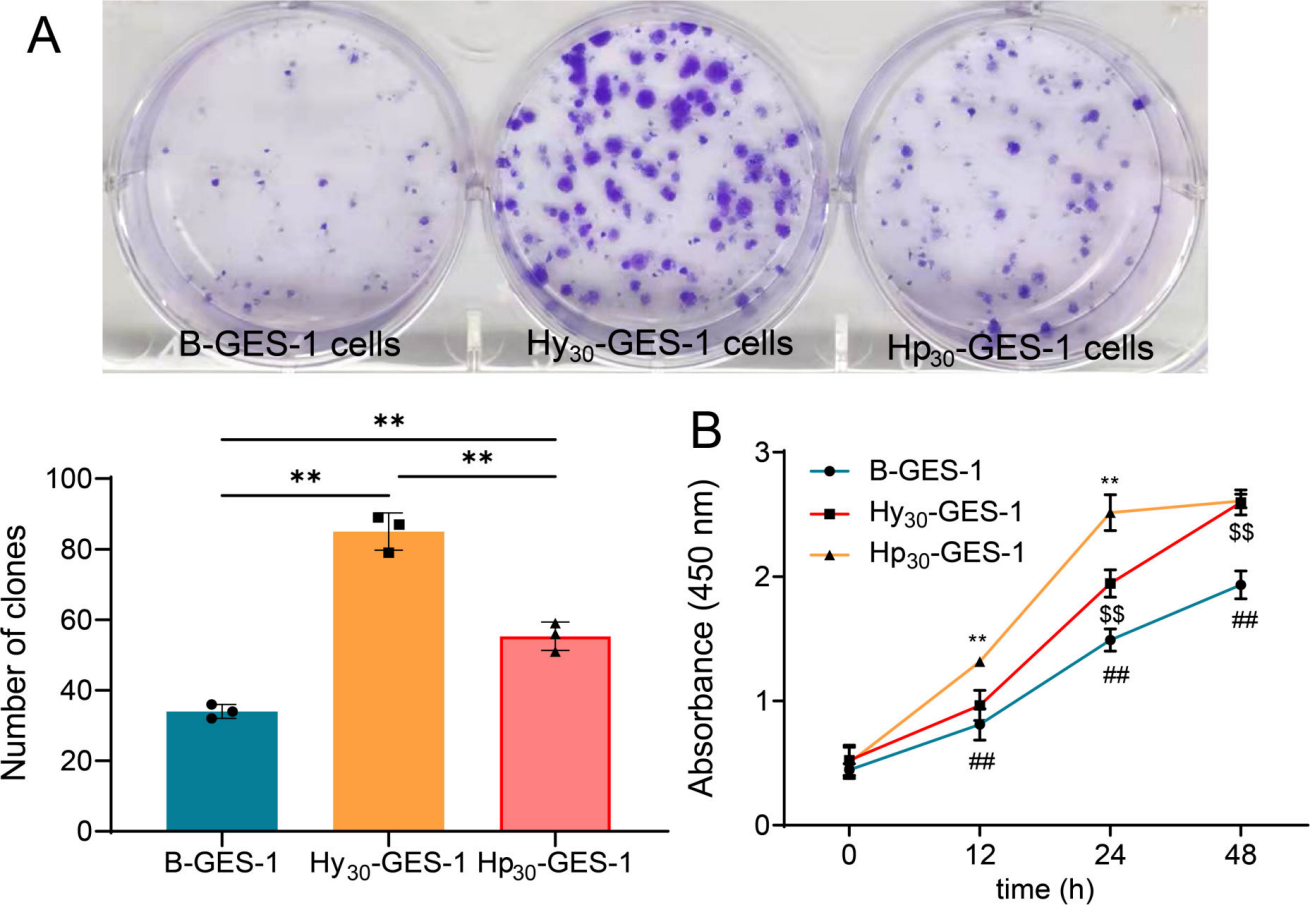
Sustained exposure to *H. pylori* under hypoxic conditions promoted cell proliferation of GES-1 cells. (**A and B**) The proliferation of Hp_30_-GES-1, Hy_30_-GES-1, and B-GES-1 cells was detected by the plate cloning assay (*n* = 3) (**A**) and CCK-8 assay (*n* = 5) (**B**). B-GES-1 vs Hy_30_-GES-1, ^$$^*P* < 0.01. Hy_30_-GES-1 vs Hp_30_-GES-1, ^**^P < 0.01. B-GES-1 vs Hp_30_-GES-1, ^##^*P* < 0.01.

To investigate the regulation of *H. pylori* on cell growth under hypoxic conditions, proliferation assays were performed. The colony formation assay revealed that Hp_30_-GES-1 cells exhibited higher activity than B-GES-1 and Hy_30_-GES-1 cells, while Hy_30_-GES-1 cells showed greater activity than B-GES-1 cells (Fig. 2A). This finding was further supported by the CCK-8 assay (Fig. 2B). These findings demonstrate that prolonged exposure to hypoxic conditions significantly amplifies the functional capacity of GES-1 cells. Additionally, *H. pylori* infection has the potential to synergistically augment the cell proliferation stimulated by hypoxia. Furthermore, the cells maintain robust activity even after long-term exposure to *H. pylori* and hypoxia.

### Sustained exposure to *H. pylori* under hypoxic conditions promotes cell migration and invasion

**Fig 3:**
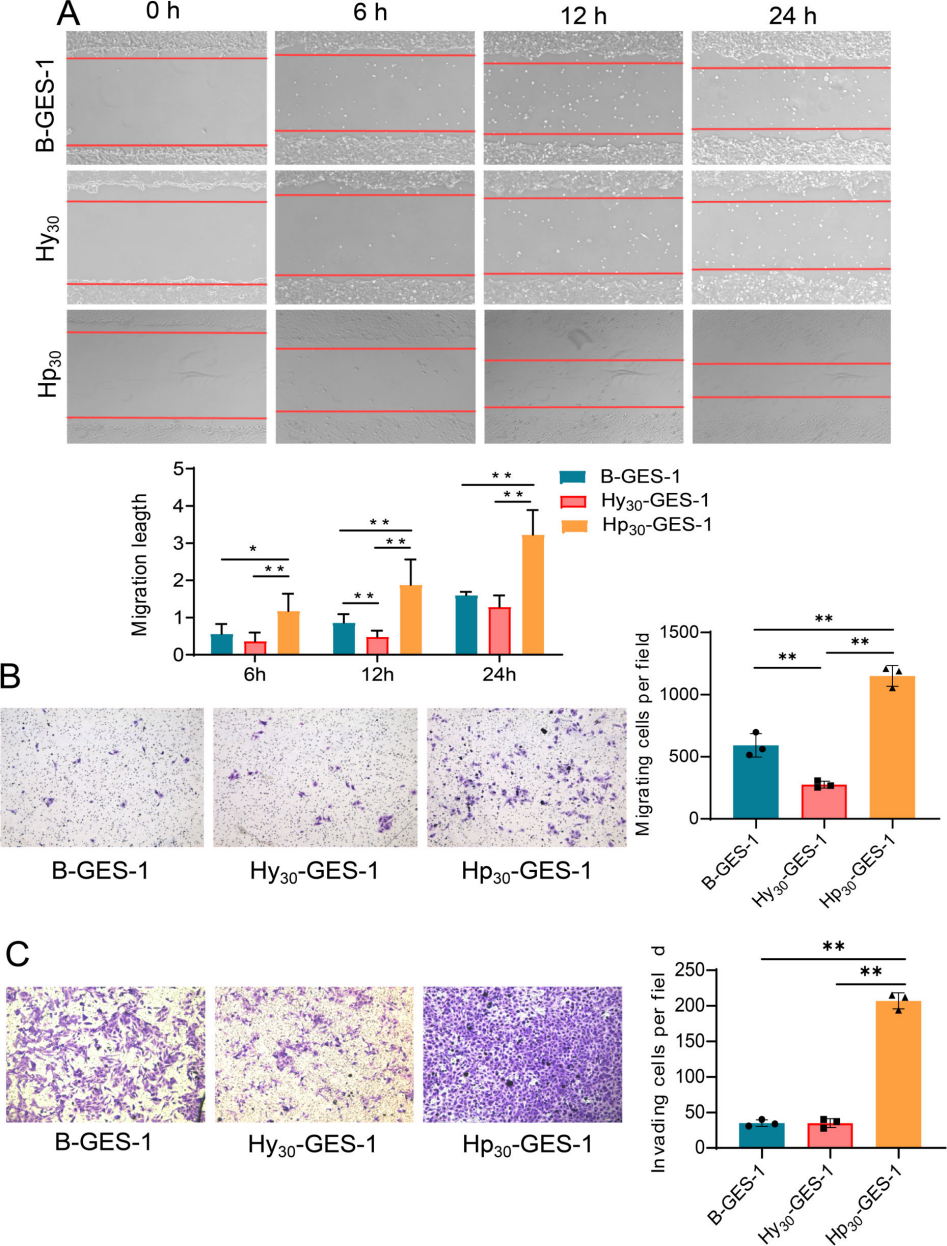
Sustained exposure to *H. pylori* under hypoxic conditions promoted migration and invasion of GES-1 cells. (**A**) The wound healing assay was used to determine the migration of Hp_30_-GES-1, Hy_30_-GES-1, and B-GES-1 cells. Red lines represent the borders of the wounds (*n* = 6). (**B and C**) Transwell migration (**B**) and Transwell invasion (**C**) assays were performed to detect the migration and invasion ability of Hp_30_-GES-1, Hy_30_-GES-1, and B-GES-1 cells. *n* = 3. *P < 0.05, **P < 0.01, and ***P < 0.05.

To investigate the regulation of gastric epithelial cell migration and invasion by *H. pylori*, wound healing and transwell assays were conducted. The wound healing assay revealed that Hy_30_-GES-1 cells had significantly inhibited migratory ability compared with B-GES-1 cells, while Hp_30_-GES-1 cells showed significantly enhanced migratory ability (Fig. 3A). The transwell migration assay yielded consistent results (Fig. 3B), indicating that long-term exposure to *H. pylori* under hypoxic conditions significantly promoted cell migration.

Regarding the cell invasion assay, long-term exposure to hypoxic conditions did not significantly affect the invasive ability of the cells. However, the invasive ability of Hy_30_-GES-1 cells was significantly enhanced compared with B-GES-1 and Hp_30_-GES-1 cells (Fig. 3C). The observed phenomenon may be attributed to the intrinsic robust invasive capacity and comparable week migratory propensity of the GES-1 cell line (18). Extended hypoxia conditions and subsequent recovery do not alter the invasive potential of GES-1 cells but rather exert an additional suppressive effect on their migratory capabilities. These results indicated that sustained exposure to *H. pylori* under hypoxic conditions promotes cell invasion, while hypoxia treatment alone does not affect the invasive ability.

### Continuous treatment with *H. pylori* under hypoxic conditions inhibits autophagy

**Fig 4:**
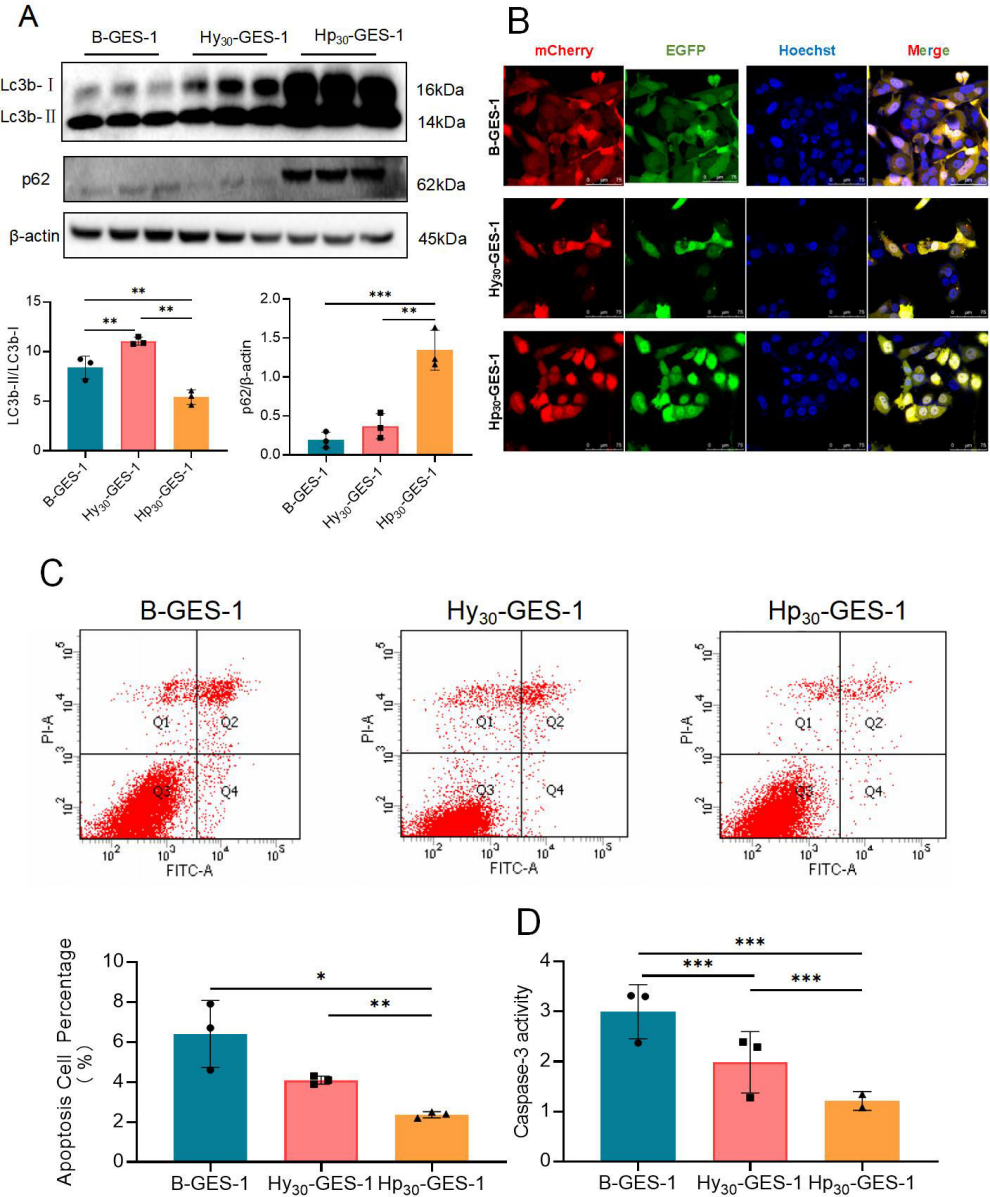
Sustained exposure to *H. pylori* under hypoxic conditions inhibited autophagy and apoptosis of GES-1 cells. (**A and B**) The expression of LC3b-II/LC3b-I (**A**) and p62 (**B**) in Hp_30_-GES-1, Hy_30_-GES-1, and B-GES-1 cells was detected by western blot. (**C**) Representative fluorescence images of autophagosomes and autolysosomes in Hp_30_-GES-1, Hy_30_-GES-1, and B-GES-1 cells using the tandem mCherry-EGFP-LC3 fusion protein assay. The autophagy flux was evaluated by the ratio of red spots to yellow spots. The yellow spots indicate autophagosomes, while the red spots indicate autolysosomes. If the phagosome and lysosome fuses normally, then the red fluorescence is greater than the yellow fluorescence. If downstream autophagy is blocked, the phagosome and lysosome cannot fuse normally, and then, yellow fluorescence is the main color visualized. (**D**) Apoptosis of Hp_30_-GES-1, Hy_30_-GES-1, and B-GES-1 cells was detected by flow cytometry. (**E**) The activity of caspase-3 in Hp_30_-GES-1, Hy_30_-GES-1, and B-GES-1 cells was detected by immunoenzyme-linked adsorption test. *n* = 3. *n* = 3. *P < 0.05, **P < 0.01, and ***P < 0.01.

Hp_30_-GES-1, Hy_30_-GES-1, and B-GES-1 cells were collected for western blot assays to detect the expression of LC3-I and LC3B-II. The magnitude of the LC3-II/LC3-I ratio can estimate the level of autophagy. The results indicated that the level of LC3-II/LC3-I in Hp_30_-GES-1 cells was significantly lower than that in both B-GES-1 and Hy_30_-GES-1 cells (Fig. 4A). Conversely, the LC3-II/LC3-I ratio in Hy_30_-GES-1 cells was upregulated compared with B-GES-1 cells (Fig. 4A). These findings demonstrated that long-term exposure to *H. pylori* under hypoxia inhibited autophagy in GES-1 cells, while hypoxic treatment without *H. pylori* promoted autophagy in gastric epithelial cells.

We also evaluated the expression of p62, which is regulated by the production and removal of autophagosomes along with LC3B-II. Western blot assays showed that the expression of p62 was increased in Hp_30_-GES-1 cells, while it remained unchanged in Hy_30_-GES-1 cells compared with B-GES-1 cells (Fig. 4B). Moreover, B-GES-1, Hy30-GES-1, and Hp30-GES-1 cells were transfected with the mCherry-EGFP-LC3 lentiviral vector to detect autophagy flux. The EGFP signal in the mCherry-EGFP-LC3 fusion protein is quenched under acidic pH in autophagolysosomes, making autophagolysosomes (GFP negative/RFP positive; red dots) and autophagosomes (GFP positive/RFP positive; yellow dots) easier to detect (Fig. 4C). In Hp_30_-GES-1 cells, the yellow puncta were more numerous than the red puncta, which contrasted with B-GES-1 and Hy30-GES-1 cells (Fig. 4C). These observations suggest that long-term treatment of *H. pylori* with hypoxia inhibits autophagy flux in GES-1 cells.

### Constant treatment of *H. pylori* under hypoxic conditions inhibits cell apoptosis

We then investigated whether *H. pylori* regulates the apoptosis of GES-1 cells under hypoxic condition. The flow cytometry analysis was conducted, and the results showed that apoptosis of Hp_30_-GES-1 cells was significantly inhibited compared with B-GES-1 and Hy_30_-GES-1 cells, while apoptosis of Hy_30_-GES-1 cells decreased compared with that inB-GES-1 cells (Fig. 4D). We also detected the activity of caspase-3, and the results further supported the aforementioned findings (Fig. 4E). These results indicate that long-term treatment of *H. pylori* under hypoxia inhibits the apoptosis of GES-1 cells.

### Sustained exposure to *H. pylori* under hypoxic conditions induces epithelial mesenchymal transition

**Fig 5:**
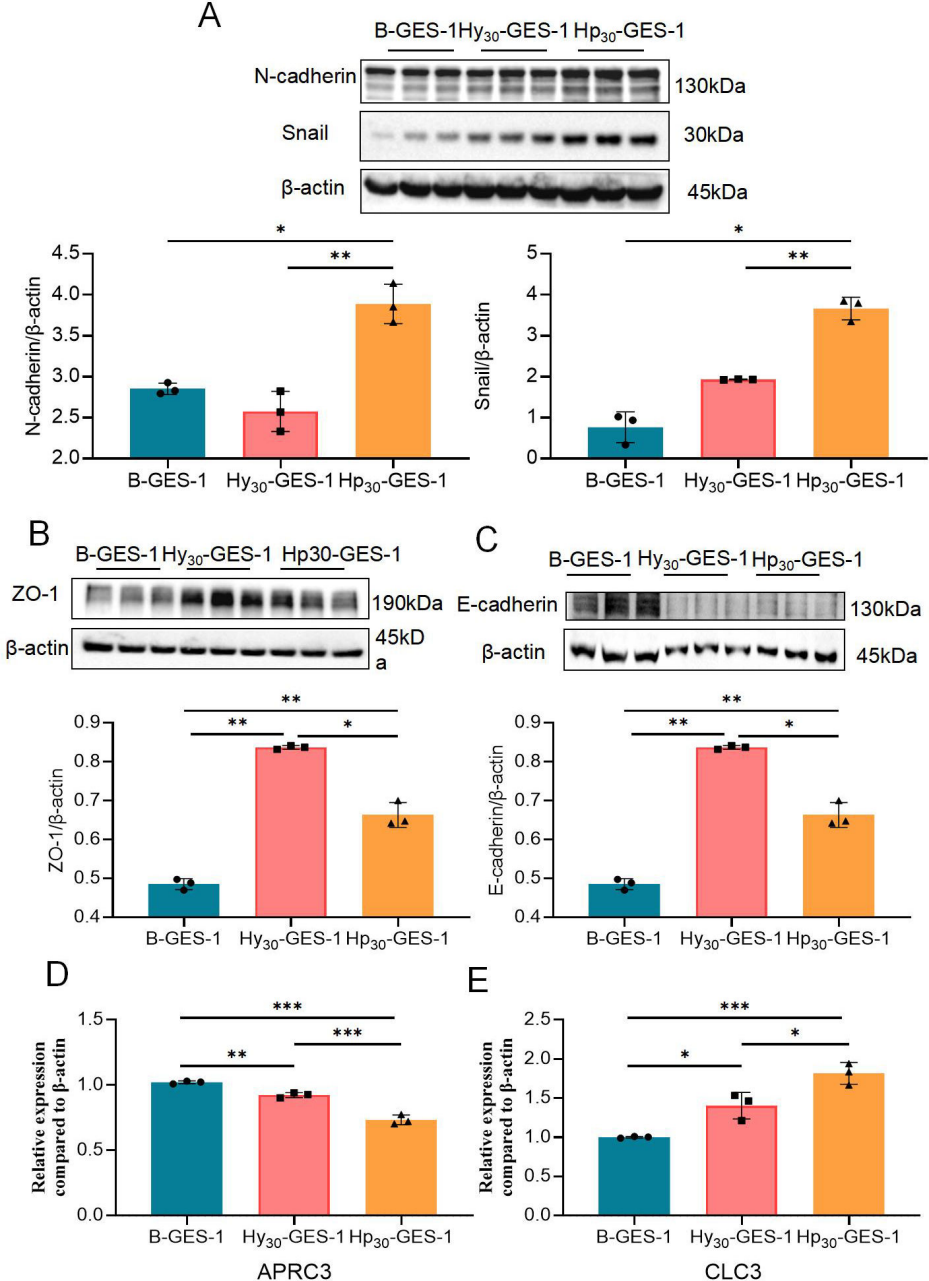
Sustained exposure to *H. pylori* under hypoxic conditions induced epithelial mesenchymal transition of GES-1 cells. (**A–D**) The expression of N-cadherin (**A**), Snail (**B**), ZO-1 (**C**), and E-cadherin (**D**) in Hp_30_-GES-1, Hy_30_-GES-1, and B-GES-1 cells wase detected by western blot. (**E**) Real-time Real-time quantitative PCR (RT-qPCR) results showing mRNA levels of *CLC3* and *ARPC3* of Hp_30_-GES-1, Hy_30_-GES-1, and B-GES-1 cells and normalized to control cells. *n* = 3. *P < 0.05, **P < 0.01, and ***P < 0.01.

Previous studies have demonstrated that epithelial mesenchymal transition (EMT)-induced carcinogenesis is a prevalent cause of various malignancies (19, 20). Therefore, we hypothesized that long-term *H. pylori* infection under hypoxia conditions could promote malignant progression. To investigate this, we assessed the expression of mesenchymal marker N-cadherin, transcription factor Snail, epithelial markers E-cadherin, and tight junction protein zonula occludens 1 (ZO-1). Our findings revealed that the expression of N-cadherin and Snail in Hp_30_-GES-1 cells was significantly higher than in both B-GES-1 and Hy_30_-GES-1 cells (Fig. 5A and B). The level of ZO-1 in Hp_30_-GES-1 cells was higher than that in B-GES-1 cells, but lower than that in Hy_30_-GES-1 cells (Fig. 5C). E-Cadherin levels in Hp_30_-GES-1 and Hy_30_-GES-1 cells were downregulated compared with B-GES-1 cells (Fig. 5D).

Furthermore, we examined the mRNA expression levels of two gastric cancer biomarkers, chloride channel-3 (*CLC-3*) and actin-related protein 2/3 complex subunit 3 (*ARPC3*) (21–23). Our results showed that the mRNA levels of *CLC-3* in Hy_30_-GES-1 cells increased significantly compared with those in B-GES-1 cells, while the *CLC-3* mRNA levels in Hp_30_-GES-1 cells were even higher than those in in Hy_30_-GES-1 cells (Fig. 5E). Additionally, *ARPC3* mRNA levels in Hp_30_-GES-1 cells decreased significantly compared with those in both control groups (Fig. 5E). These findings suggest that long-term stimulation by *H. pylori* under hypoxia induces EMT and contributes to the malignant transformation of gastric epithelial cells.

### *H. pylori* inhibit cell autophagy and apoptosis under hypoxia through the Nod1 receptor pathway

In our previous study, we screened signaling pathways involved in *H. pylori*-induced gastric cancer based on the TCGA database (https://cancergenome.nih.gov/) and conducted cell experiments involving long-term stimulation of *H. pylori* lysate (24). We discovered that persistent treatment with *H. pylori* lysate regulates autophagy and apoptosis of gastric epithelial cells through the *Nod1-RIP2-MAPK/ERK-FOXO4* and Nod1-RIP2-NF-κB pathways, respectively. Therefore, in this study, we investigated the changes in the Nod1 receptor pathway.

**Fig 6:**
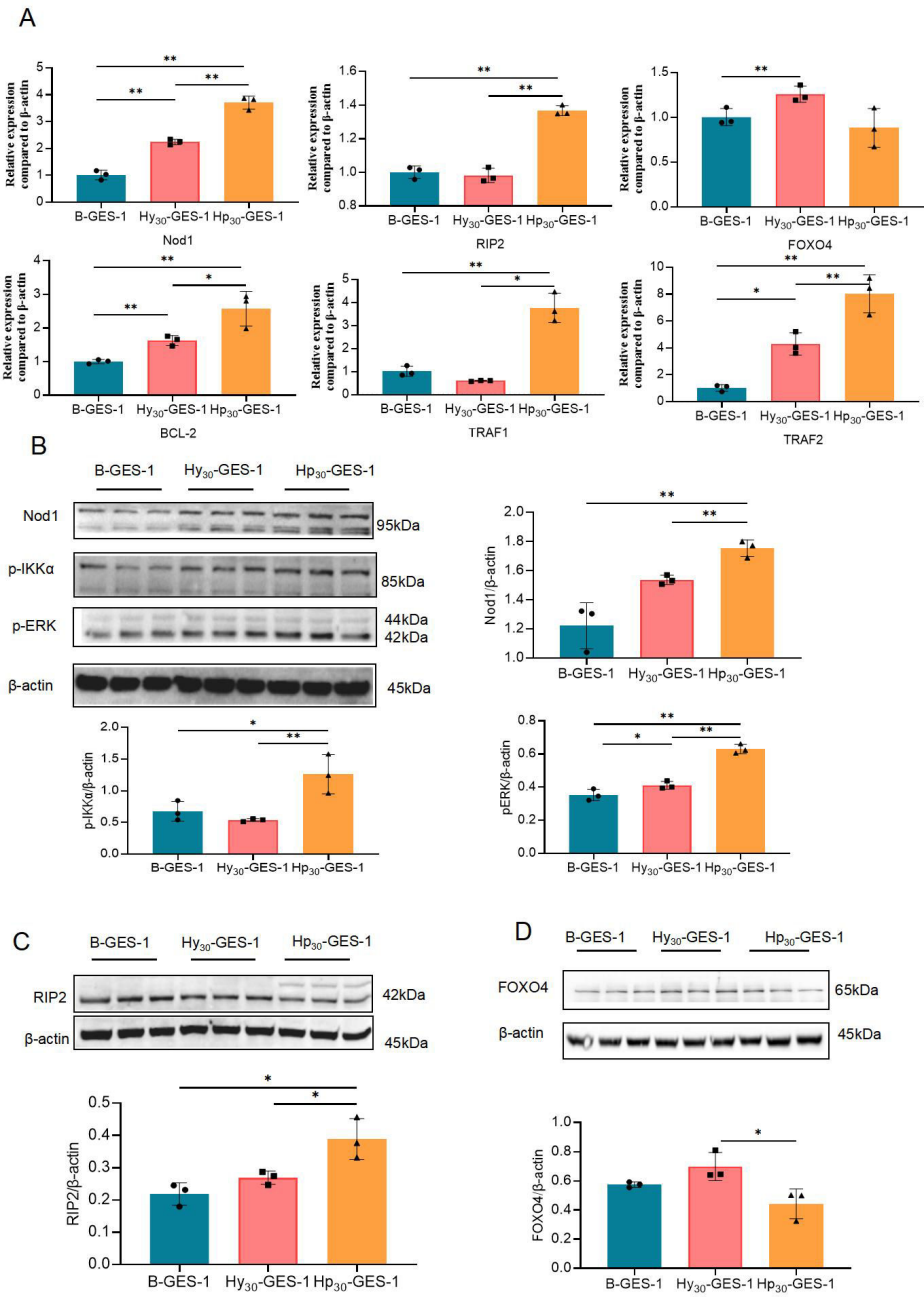
Pathways related to the inhibition of autophagy and apoptosis of GES-1 cells sustained exposure to *H. pylori* under hypoxic conditions. (**A**) mRNA expression of *Nod1*, *RIP2*, *FOXO4*, *BCL-2*, and *TRAF1/2* in Hp_30_-GES-1, Hy_30_-GES-1, and B-GES-1 cells tested by real-time qPCR. (**B–G**) Expression of Nod1 (B), RIP2 (**C**), p-IKKα (**D**), p-ERK (**E**), and FOXO4 (**F**) measured by western blot. *n* = 3. *P < 0.05 and **P < 0.01.

We evaluated the mRNA and protein expression levels of key genes involved in the *Nod1-RIP2-MAPK/ERK-FOXO4* and *Nod1-RIP2-NF-κB* pathways. Our results demonstrated that the mRNA and protein expression levels of Nod1 and receptor-interacting protein 2 (RIP2) were significantly upregulated in Hp_30_-GES-1 cells compared with B-GES-1 and Hy_30_-GES-1 cells (Fig. 6A through C). Additionally, the phosphorylation level of IKKα and ERK increased in Hp_30_-GES-1 cells (Fig. 6D), while the mRNA level of FOXO4 was inhibited in Hp_30_-GES-1 cells without statistically significant differences (Fig. 6A). The protein expression of FOXO4 decreased significantly compared with B-GES-1 and Hy_30_-GES-1 cells (Fig. 6E). Moreover, the mRNA expression levels of TRAF1/2 and BCL-2 in Hp_30_-GES-1 cells were significantly higher than in Hy_30_-GES-1 and B-GES-1 cells (Fig. 6A). These findings indicate that long-term stimulation of *H. pylori* under hypoxic conditions regulates the function of GES-1 cells through the Nod1 receptor pathway.

### Long-term infection of *H. pylori* under hypoxia inhibits autophagy and apoptosis through the Nod1 receptor pathway *in vivo*

**Fig 7:**
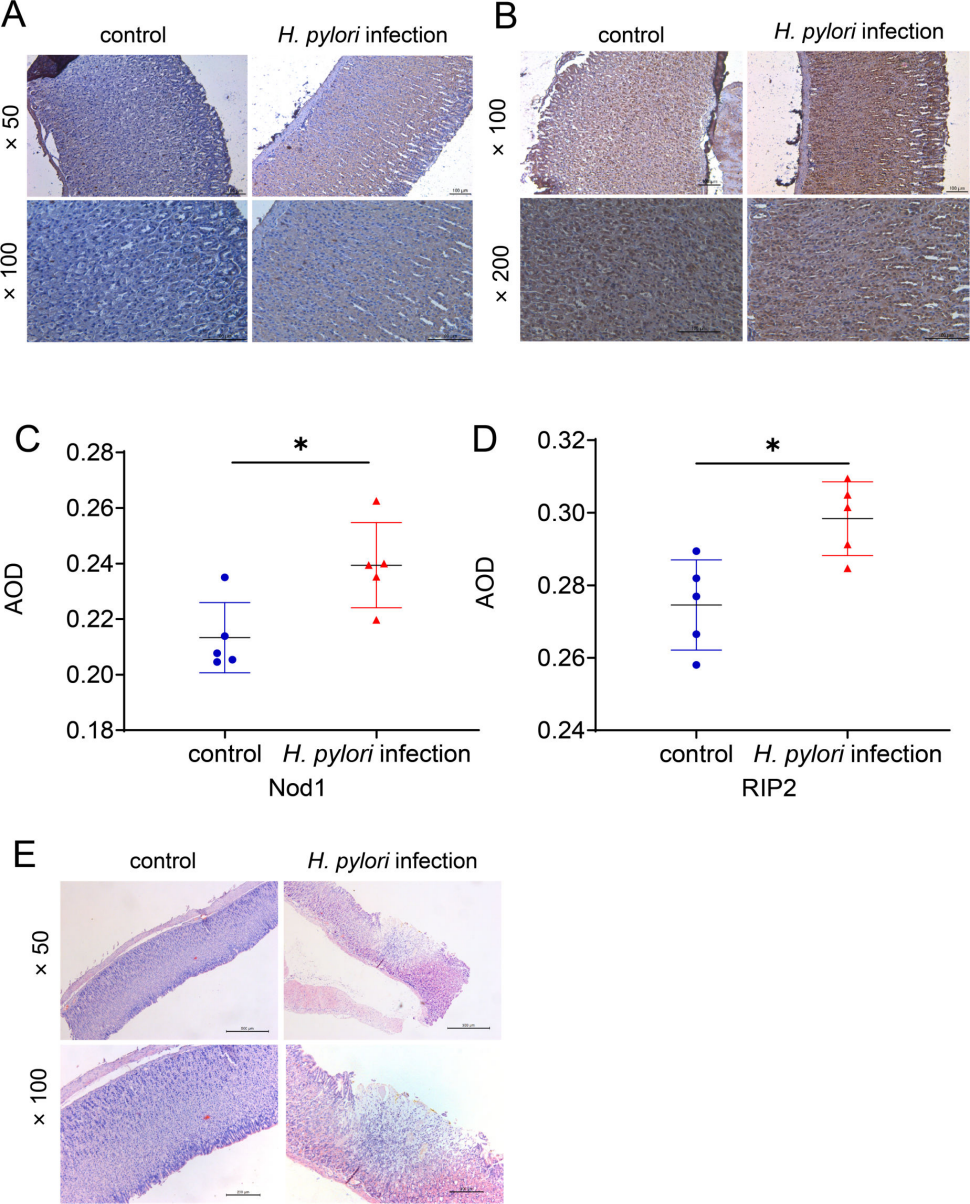
Sustained infection of *H. pylori* under hypoxia inhibited autophagy and apoptosis through the Nod1 receptor pathway *in vivo*. (**A–D**) Immunohistochemistry was performed to detected the expression of Nod1 (**A and C**) and RIP2 (**B and D**) in Hp_30_-GES-1, Hy_30_-GES-1, and B-GES-1 cells. (**E**) HE staining revealing the pathology of Mongolian gerbil stomach samples. Control: gerbils (*n* = 5) were administered sterile saline solution by gavage as a control group. *H. pylori* infected: gerbils (*n* = 5) were orally infected with *H. pylori* ATCC 43504 strain solution. Representative histologic images from *H. pylori*-infected gerbils at original magnification ×50 and ×100. *n* = 5. *P < 0.05 and **P < 0.01.

We have previously confirmed that long-term infection of *H. pylori* inhibits autophagy and apoptosis of gastric epithelial cells *in vivo* (24). In this study, we investigated the influence of *H. pylori* infection on autophagy and apoptosis *in vivo*. We infected gerbils with the *H. pylori* ATCC 43504 strain for 85 weeks and collected gastric tissues. Immunohistochemistry was performed to detect the expression of Nod1 and RIP2 in gastric epithelial cells. The results showed a remarkable increase in the expression levels of Nod1 and RIP2 in gerbils continuously exposed to *H. pylori* infection compared with the control group (Fig. 7A through D). Furthermore, pathological analysis revealed that *H. pylori* infection induced dysplasia in the gastric mucosa of gerbils (Fig. 7E). These findings provide additional evidence supporting the involvement of *H. pylori* in facilitating the malignant transformation of gastric epithelial cells. All the data verify the key roles of the Nod1 receptor pathway in *H. pylori-*induced inhibition of autophagy and apoptosis *in vivo*.

## DISCUSSION

*H. pylori* infects about half of the global population and is the most important cause of cancer infection, accounting for approximately 90% of new cases of gastric cancer worldwide (25–28). Currently, numerous studies have elucidated the pathogenic mechanisms of *H. pylori* both *in vivo* and *in vitro*. However, it is noteworthy that most *in vitro* experiments are conducted under normal pO_2_ conditions (20.265 kPa). This approach falls short of accurately replicating the growth environment of gastric epithelial cells *in vivo*, primarily due to the considerably lower oxygen levels present in the human stomach (pO_2_ = 8 kPa) (16, 17, 29). Furthermore, as a microaerophile, *H. pylori* is greatly influenced by oxygen concentration, and this, in turn, affects its form and pathogenicity (14, 30). *H. pylori* cultured under a 7% O_2_ concentration exhibit the bacillary form, whereas the cells cultured under aerobic conditions adopt the coccoid form (14, 30). The conversion from the bacillary to the coccoid form leads to a significant reduction of bacterial toxicity (31). Certain characteristics displayed by *H. pylori* cultivated under microaerobic conditions more closely resemble the physiology of organisms grown in the human gastric mucosa (32). Therefore, in this study, we established a cell line infected with *H. pylori* under hypoxic conditions, simulated the gastric environment, and explored the pathogenic mechanism of persistent *H. pylori* infection under hypoxic conditions.

Cell migration and invasion play crucial roles in tumor development., *H. pylori* infection has been demonstrated to enhance the migratory and invasive capabilities of gastric cells in a CagA-dependent manner (33). Cells infected with CagA^+^ *H. pylori*, as compared with CagA- H. pylori, exhibit upregulation of miR-543, a potent inducer of cell migration and invasion (34). Additionally, studies have shown that CagA protein activates the NLRP3 inflammasome, promoting migration and invasion of gastric cancer cells (35). Moreover, previous research by Guo et al. has indicated that hypoxia contributes to the migration and invasion of MGC-823 cells (36). Notably, AGS cells demonstrated heightened invasive capabilities under a hypoxic microenvironment (37). In our research, we observed that prolonged hypoxic stimulation of *H. pylori* significantly enhanced the migration and invasion abilities of GES-1 cells, consistent with previous reports (33–35). However, interestingly, prolonged hypoxic stimulation did not enhance the migration and invasion of GES-1 cells compared with the control group. This lack of enhancement could be attributed to unique characteristics of different cell lines and variations in their origins. In general, our results support the notion that prolonged *H. pylori* stimulation contributes to cell migration and invasion under a hypoxic condition more similar to the *in vivo* environment.

Autophagy plays a pivotal role in the survival and carcinogenesis of *H. pylori*. Prior investigations have documented that *H. pylori* infiltrates gastric epithelial cells, disrupts autophagic lysosomal acidification, and evades them to bolster survival and colonization (38–40). Suppression of *H. pylori*-induced autophagy fosters the progression of gastric cancer by impairing DNA damage repair and inducing genome instability (41). In our study, we validated the inhibition of cellular autophagic flux during both hypoxia and prolonged *H. pylori* stimulation. This inhibition potentially contributes to the prolonged survival, proliferation, and immune evasion of bacteria.

The balance between cell proliferation and apoptosis is intricately regulated, and disruption of this balance can lead to the malignant transformation of cells. Various components produced by *H. pylori* have regulatory effects on the proliferation of gastric epithelial cells (42). *H. pylori* toxins, such as UreA and CagA, have been confirmed to promote the proliferation of gastric epithelial cells and increase the risk of cancer, as demonstrated in a guinea pig model infected with *H. pylori* (43). Additionally, *H. pylori* infection has been shown to inhibit apoptosis in gastric epithelial cells (44, 45). Our research revealed that prolonged exposure to *H. pylori* under a hypoxic environment stimulates the proliferation of gastric epithelial cells, hinders cell apoptosis, and induces epithelial-to-mesenchymal transition. These results once again confirmed the promotive role of *H. pylori* infection in carcinogenesis.

Our findings suggest that prolonged exposure to *H. pylori* in a hypoxic environment not only inhibits autophagy and apoptosis but also significantly enhances the migration and invasion capabilities of GES-1 cells. Meanwhile, hypoxia alone does not exert the same effects on cell migration and invasion as those observed in *H. pylori*-infected cells. It is important to note that *H. pylori* thrives in the microaerobic environment of the human gastric mucosa (2). Notably, under hypoxic conditions, *H. pylori* assumes a bacillary form, which is more virulent and better equipped to colonize the gastric mucosa (14). Therefore, our study underlines the synergistic effects of hypoxic conditions and *H. pylori* infection, providing a more accurate simulation of the *in vivo* gastric environment.

**Fig 8:**
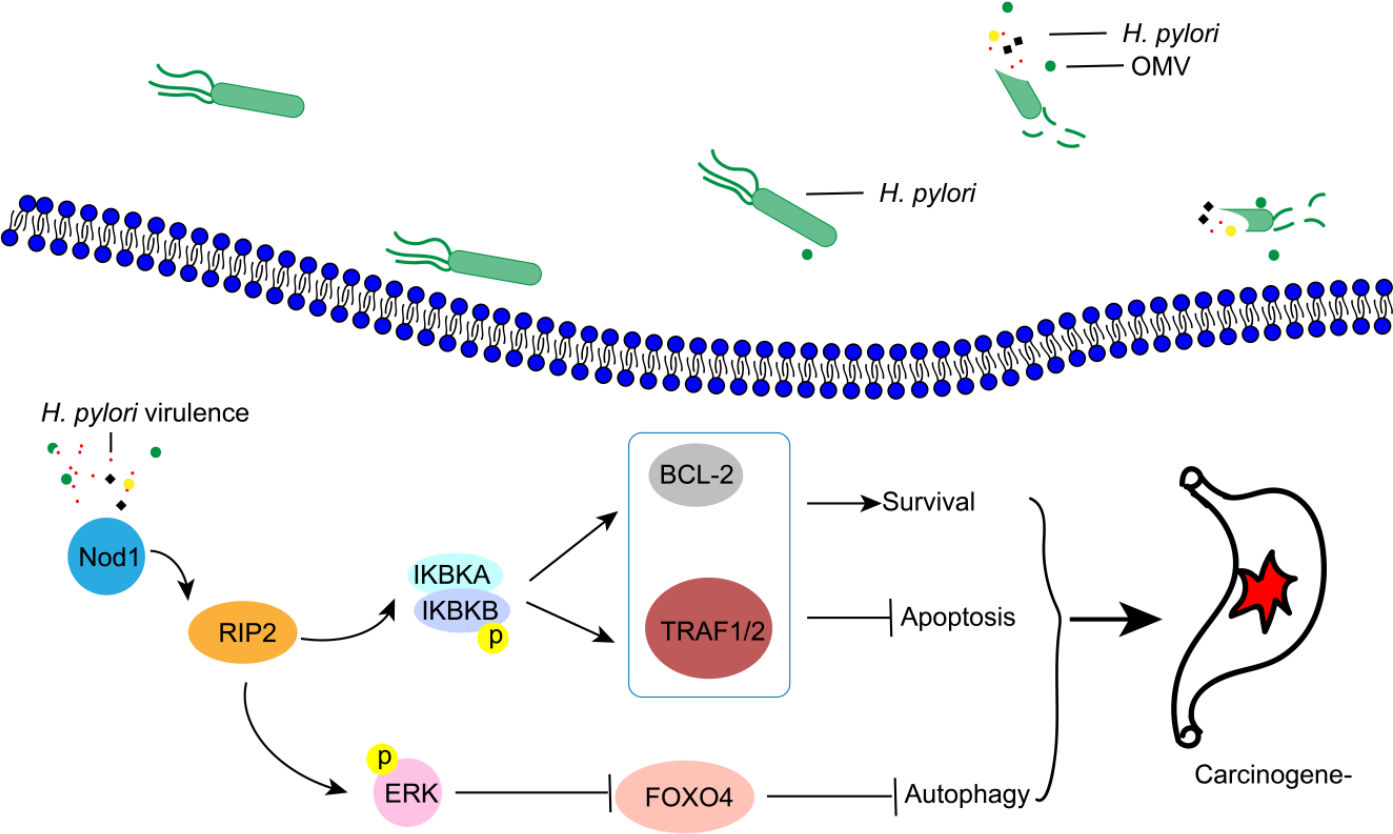
A model of the Nod signal pathway.

The Nod1 receptor pathway plays a crucial role in the regulation of host cell functions in response to *H. pylori* (46, 47). Nod1 receptors recognize *H. pylori’s* peptidoglycan, thereby modulating the autophagy and apoptosis of host cells (48). In our previous studies, we have identified the involvement of the Nod1 pathway in regulating gastric epithelial cell autophagy and apoptosis during prolonged stimulation with *H. pylori* lysate (24). Therefore, in this study, we investigate the role of the Nod1 pathway in regulating host cell functions under long-term hypoxic stimulation by *H. pylori*, and the results were consistent with our previous findings. Furthermore, existing literature reports suggest that CagA regulates autophagy and apoptosis of gastric epithelial cells through the NF-κB and MAPK/ERK pathways (45, 49). Based on this, we hypothesize that during prolonged *H. pylori* stimulation, CagA is internalized into host cells via the T4SS and then binds to SHP-2, thereby regulating downstream NF-κB and MAPK/ERK pathways. Other bacterial toxins such as VacA and peptidoglycan are delivered into host cells through OMV. These toxins modulate the downstream NF-κB and MAPK/ERK pathways through Nod1 recognition. Collectively, all these pathways work together to promote tumorigenesis (Fig. 8).

In summary, our study provides insights into the synergistic effects of *H. pylori* infection and hypoxic conditions on the function of gastric epithelial cells. Prolonged exposure to *H. pylori* under hypoxia significantly enhanced cell migration, invasion, and epithelial mesenchymal transition, while inhibiting autophagy and apoptosis. These findings underscore the importance of the Nod1 signaling pathway in mediating the inhibitory effects of *H. pylori* on autophagy and apoptosis. The establishment of an *H. pylori*-infected cell line under hypoxic conditions provides a more accurate cell model for studying the pathogenic mechanisms of *H. pylori* in gastric carcinogenesis.

## SUPPLEMENTAL MATERIAL

Tables S1 spectrum.00311-24-s0001.docx Nucleotide sequences of primers used for RT-PCR reactions.

ASM does not own the copyrights to Supplemental Material that may be linked to, or accessed through, an article. The authors have granted ASM a non-exclusive, world-wide license to publish the Supplemental Material files. Please contact the corresponding author directly for reuse.
