# Supplementary material for: Hypoxia exacerbates the malignant transformation of gastric epithelial cells induced by long-term H. pylori infection
Source: Microbiol Spectr. 2024 Jun 25;12(8):e00311-24. doi: 10.1128/spectrum.00311-24 (PMC11302036; doi:10.1128/spectrum.00311-24)
Supplement: Tables S1 — Nucleotide sequences of primers used for RT-PCR reactions. [file spectrum.00311-24-s0001.docx]

Supplementary Table 1. Nucleotide sequences of primers used for RT-PCR reactions

| Gene | Forward | Reverse |
| --- | --- | --- |
| Nod1 | 5'-TGGTGGCCAAGTGATTGTAAGT-3' | 5'-TCTGTAATCGCCGCCACAAT-3' |
| RIP2 | 5'-CGCTGCTCGACAGTGAAAGA-3' | 5'-TTCAGGCTCATTGCAAATTCCC-3' |
| BCL-2 | 5'-GGTGGGGTCATGTGTGTGG-3' | 5'-CGGTTCAGGTACTCAGTCATCC-3' |
| TRAF1 | 5'-TAGGCGGTGGCGGAGG-3' | 5'-ATCCCCTGGATGGTGACTGA-3' |
| TRAF2 | 5'-CTCGGTGTGAGCAAGTGGAC-3' | 5'-GCTAGCTGCAGCCATGAGAG-3' |
| FOXO4 | 5'-CCAGAGATCGCTAACCAGCC-3' | 5'-CTTTCAATGGCCTTTTCCCCC-3' |
| CLC3 | 5'-TCTGAGCAGCTGTTCCATAGAG-3' | 5'-GCTGCCTCCATTTGTCATTGT-3' |
| ARPC3 | 5'-TGCCCCCAGAGAGACAAAAGA-3' | 5'-TGCACTTTTGCAGTTTCTTCAGAC-3' |
